# A prospective investigation of injury incidence and injury risk factors among army recruits in military police training

**DOI:** 10.1186/1471-2474-14-32

**Published:** 2013-01-17

**Authors:** Joseph J Knapik, Bria Graham, Jacketta Cobbs, Diane Thompson, Ryan Steelman, Bruce H Jones

**Affiliations:** 1 US Army Institute of Public Health, Portfolio of Epidemiology and Disease Surveillance, ATTN: MCHB-IP-DI, 1570 Stark Road, Aberdeen Proving Ground, MD, 21010, USA; 2 Medical University of South Carolina, Department of Obstetrics and Gynecology, Charleston, SC, USA; 3 AIDS Resource Center of Wisconsin, Milwaukee, WI, 53203, USA

**Keywords:** Age, Smoking, Exercise, Physical activity, Prior injury, Menstrual cycle, Pregnancy

## Abstract

**Background:**

United States Army military police (MP) training is a 19-week course designed to introduce new recruits to basic soldiering skills, Army values and lifestyle, and law enforcement skills and knowledge. The present investigation examined injury rates and injury risk factors in MP training.

**Methods:**

At the start of training, 1,838 male and 553 female MP recruits were administered a questionnaire containing items on date of birth, height, weight, tobacco use, prior physical activity, injury history, and menstrual history. Injuries during training were obtained from electronic medical records and the training units provided data on student graduation and attrition.

**Results:**

Successfully graduating from the course were 94.3% of the men and 83.7% of the women. Experiencing at least one injury during training were 34.2% of the men and 66.7% of the women (risk ratio (women/men) = 1.95, 95% confidence interval = 1.79-2.13). Recruits were at higher injury risk if they reported that they were older, had smoked in the past, or had performed less frequent exercise or sports prior to MP training. Men were at higher injury risk if they reported a prior injury and women were at higher risk if they reported missing at least six menstrual cycles in the last year or had previously been pregnant.

**Conclusion:**

The present investigation was the first to identify injury rates and identify specific factors increasing injury risk during MP training.

## Background

United States (US) Army Basic Combat Training is a 10-week course designed to develop basic soldiering skills and introduce a new Army recruit into Army values and lifestyle. Most US soldiers complete basic training first and then move on to Advanced Individual Training where they learn their military occupational specialty with a new group of soldiers at another location. However, some military occupational specialties require soldiers to train as a cohort and move directly from basic training into their occupational specialty training with the same group of soldiers and some overlap between Basic Combat Training and occupational specialty training. This is called One-Station Unit Training (OSUT). One type of OSUT is the 19-week course conducted for the military police (MP). Like basic training, the first 10 weeks are devoted to the development of basic soldiering skills in which the recruit is very physically active. There is almost daily physical training in the morning in addition to marksmanship training, periodic road marches, confidence/obstacle course negotiation, high tower operations, team and individual movement exercises, land navigation, and other physical activities. The latter weeks are devoted to training more specific to the occupational specialty including unarmed self-defense and suspect apprehension, civil disturbance training, law and order operations, special weapons instruction, area security exercises, intelligence operations, enemy prisoner of war/civilian internee exercises, use and set-up of tactical control points, convoy and patrol operations, search and seizure operations, and use of levels of force.

Recruits in basic training and OSUT perform a large volume of physical training and physical activity and because of this they are at risk of injury [1]. A number of previous studies have examined injury rates in basic training [2-5] and these investigations indicate that recruits have one of the highest injury rates of any group in the military [6]. Only one previous investigation has examined OSUT training and that was in infantry training that involved no women [7]. The purpose of the present study was to examine injury rates and injury risk factors among male and female recruits in MP OSUT.

## Methods

Participants were 1,838 male and 553 female recruits in MP OSUT at Fort Leonard Wood, Missouri. These recruits were from 14 separate training companies in two battalions that began training between 4 May 2010 and 25 February 2011 and completed training between 16 September 2010 and 7 July 2011. None of the MP OSUT companies involved in “Exodus” were included. Exodus was a two-week period over the late December and early January period when no training was conducted and the recruits were allowed to return to their homes. The study protocol was reviewed and approved by the Human Subjects Protection Office at the US Army Institute of Public Health as a public health practice project.

### Procedures

Recruits completed a lifestyle questionnaire within the first week of training. This questionnaire contained items on date of birth, height, weight, tobacco use, prior physical activity, injury history, and menstrual history.

Injury data were obtained from the Defense Medical Surveillance System of the Armed Forces Health Surveillance Center (AFHSC). The AFHSC regularly compiles data on ambulatory (outpatient) encounters occurring within military treatment facilities, as well as those occurring outside these facilities (civilian care) and paid for by the US Department of Defense. A list of recruits from the units being evaluated and the dates of their training cycles were provided to the AFHSC. The AFHSC returned visit dates and International Classification of Diseases, Revision 9, Clinical Modification (ICD-9) codes for all outpatient medical visits during the training cycle timeframe. Five injury indices were calculated from the data provided by the AFHSC. These indices were the Installation Injury Index, the Modified Installation Injury Index, the Training-Related Injury Index, the Comprehensive Injury Index (CII), and the Overuse Injury Index. These indices included specific ICD-9 codes, as described previously [8]. The Installation Injury Index and Training-Related Injury Index were developed by personnel at the AFHSC. The Installation Injury Index has been used to compare overall injury rates (acute and overuse) among military posts and is reported on a monthly basis at the AFHSC website (http://afhsc.army.mil). The Training-Related Injury Index is limited to lower extremity overuse injuries and has been used to compare injury rates among Army Basic Combat Training locations. The Modified Installation Injury Index, CII, and Overuse Injury Index were developed by personnel in the Injury Prevention Program at the Army Institute of Public Health. The Modified Installation Injury Index captures a greater number of injuries than the Installation Injury Index, including more overuse-type injuries. The CII captures all ICD-9 codes related to injuries defined as physical damage to the body as a result of an energy exchange [9]. The Overuse Injury Index captures the subset of musculoskeletal injuries presumably resulting from cumulative microtrauma (overuse injuries) such as stress fractures, stress reactions, tendonitis, bursitis, fasciitis, arthralgia, neuropathy, radiculopathy, shin splints, synovitis, sprains, strains, and musculoskeletal pain (not otherwise specified). The CII was the primary outcome measure in this report.

Recruits that attrited from training, as well as the date and reason were provided by the training companies. These data were verified from information in the Directorate of Human Resources, Trainee Student Processing Branch at Fort Leonard Wood and from the Resident Individual Training Management System. Attrition could have been due to discharge from service or recycling. Discharges were recruits who were not suitable for service in the Army and were formally released from their service commitment. A discharge may have been due to a medical condition that existed prior to service or developed during training, or for a non-medical reason. Non-medical discharges were generally due to the inability of the recruit to adapt to the military environment because of lack of ability (could not adequately perform critical military tasks) or for psychosocial reasons (lack of motivation, inability to follow orders, personality problems, commission of serious offenses). A recycle was a recruit who needed additional training to complete training requirements and was sent to another unit to complete this training. Recycles were not followed once they left their initial training unit.

### Data analysis

Age was calculated from the date of birth to the date of the start of training. Body mass index (BMI) was calculated as weight/height^2^ obtained from the questionnaire [10]. Cumulative injury incidence was calculated as the number of recruits with ≥1 injury/the total number of recruits X 100%. Injury incidence rate was calculated as number of recruits with ≥1 injury/the total number of recruits X number of days in training (injuries/1,000 person-days). The Open Epidemiological Calculator [11] was used to obtain comparisons (risk ratios, rate ratios and 95% confidence intervals (95%CI)) between men and women on attrition and injury variables.

Other analyses were performed using the Statistical Package for the Social Sciences (SPSS), Version 18.0. Cox regression (survival analysis) was used to examine the association between the time to the first CII injury and other potential injury risk factors from the questionnaire. Once a recruit had an injury, his or her contribution to time in training was terminated (censored). Those who attrited from training had their time censored at the day they left training, unless their time had already been censored as the result of an injury. All potential risk factors were entered into the regression models as categorical variables. Continuous variables were converted to categorical variables based on recommendations from the literature or findings from previous basic training investigations [5,6,12]. Age was separated into 3 groups (<20.0, 20.0-24.9, ≥25.0 years). BMI was separated into 3 groups (<25.0, 25.0-29.9 and ≥30 kg/m^2^) as recommended by the National Institute of Health [13]. Physical activity questions were categorized based on recommended activity levels specified by the American College of Sports Medicine [14]. For all Cox regressions, simple contrasts were used, comparing the injury hazard at a baseline stratum of a variable (defined with a hazard ratio (HR) of 1.00) with other strata of the same variable. Variables were included in a multivariate backward stepping Cox regression if they achieved p < 0.10 in the univariate analyses [15]. Multivariate Cox regressions established the association between a variable and injury risk with other significant injury risk factors included. Multivariate Cox regression requires complete data on all included cases so that any cases with without complete data are eliminated from the analysis.

## Results

The mean ± standard deviation age, height, weight, and BMI of the male recruits was 20.6 ± 3.0 years, 178 ± 7 cm, 78 ± 12 kg, and 24.6 ± 3.3 kg/m^2^, respectively. Among female recruits, these values were 20.5 ± 2.8 years, 164 ± 7 cm, 63 ± 8 kg, and 23.5 ± 2.6 kg/m^2^, respectively.

Table 1 shows the number and proportion of recruits who graduated and attrited from training. Compared to those who graduated, women were 3.13 (95%CI = 2.31-4.23) times more likely to be discharged (all causes) than men, and 2.75 (95%CI = 1.42-5.33) times more likely to be recycled than men.

**Table 1:** Graduation and Attrition in MP training

**Final status**	**Men**		**Women**	
	**N**	**Proportion (%)**	**N**	**Proportion (%)**
Graduated	1734	94.3	463	83.7
Medical Discharge	26	1.4	40	7.2
Other Discharge	53	2.9	33	6.0
Recycle	20	1.1	15	2.7
AWOL^a^	1	0.1	0	0.0
Unknown	4	0.2	2	0.4

^a^AWOL = absent without leave.

Table 2 shows the injury incidence and injury incidence rates for each of the injury indices and compares the men and women. Women were more likely to be injured than men and the two overuse injury indices (Overuse Injury Index and Training-Related Injury Index) showed larger gender differences than the other injury indices. The total amount of time in training for all recruits was 241,878 days for the men and 71,630 days for the women.

**Table 2:** Male and Female injury incidence and injury Incidence rates in MP training

**Injury Index**	**Injury incidence (%)**		**Risk ratio – Women/Men (95%CI)**	**Injury incidence rate (injuries/1,000 person-days)**		**Rate ratio – Women/Men (95%CI)**
	**Men**	**Women**		**Men**	**Women**	
Installation Injury Index	30.3	62.7	2.07 (1.88-2.28)	2.30	4.84	2.10 (1.84-2.41)
Modified Installation Injury Index	33.4	66.4	1.99 (1.82-2.17)	2.53	5.12	2.02 (1.78-2.30)
Overuse Injury Index	23.6	58.4	2.47 (2.22-2.76)	1.79	4.51	2.51 (2.18-2.90)
Training-Related Injury Index	17.8	51.2	2.88 (2.53-3.27)	1.35	3.96	2.92 (2.49-3.43)
Comprehensive Injury Index	34.2	66.7	1.95 (1.79-2.13)	2.60	5.17	1.99 (1.75-2.26)

Table 3 displays the association between CII injury risk and the variables under investigation. Not all recruits answered all questions so the sample sizes for each variable are shown. For both men and women, higher injury risk was associated with older age, having smoked ≥100 cigarettes in the past, less frequent exercise or sports activity, a shorter period of running/jogging prior to OSUT, and a shorter period of weight training prior to OSUT. For men, injury risk was also associated with a younger age for the onset of smoking, more days of smoking, more cigarettes per day, a self-rating of less physical activity prior to OSUT, and a prior injury, especially if that prior injury restricted training for a week or more or the recruit had not totally recovered from the injury. Among the women, risk was generally elevated at similar strata of these variables but the magnitudes of the risks were much smaller than that for the men. Among the women, injury risk was also associated with having gone ≥ 6 months without a menstrual cycle in the last year and having been pregnant.

**Table 3:** Univariate associations between questionnaire variables and injury risk among MP recruits

**Variable**	**Strata**	**Men**			**Women**		
		**N**	**Hazard ratio (95% CI)**	**p-value**	**N**	**Hazard ratio (95% CI)**	**p-value**
Age	<20.0 years	1080	1.00	Referent	325	1.00	Referent
	20.0-24.9 years	623	1.21 (1.04-1.46)	0.01	195	1.25 (1.00-1.55)	0.05
	25.0-29.9 years	90	1.25 (0.87-1.78)	0.23	23	1.21 (0.72-2.05)	0.47
	≥30.0 years	38	2.17 (1.41-3.33)	<0.01	10	2.99 (1.47-6.07)	<0.01
Body Mass Index	<25.0 kg/m^2^	1048	1.00	Referent	401	1.00	Referent
	25.0-29.9 kg/m^2^	658	1.13 (0.96-1.33)	0.15	145	0.91 (0.72-1.15)	0.42
	≥30 kg/m^2^	132	1.05 (0.77-1.43)	0.77	5	0.96 (0.31-3.01)	0.95
Smoked ≥100 Cigarettes in Life	No	1273	1.00	Referent	404	1.00	Referent
	Yes	563	1.37 (1.17-1.61)	<0.01	149	1.28 (1.00-1.60)	0.05
Age Started Smoking	Never	932	1.00	Referent	316	1.00	Referent
	<13 years	72	1.54 (1.07-2.23)	0.02	19	1.37 (0.78-2.40)	0.27
	13-16 years	520	1.18 (0.99-1.42)	0.07	134	1.05 (0.82-1.34)	0.72
	≥17 years	314	1.05 (0.83-1.31)	0.69	84	1.09 (0.81-1.46)	0.57
Days Smoked in 30 Days Before OSUT	None	1368	1.00	Referent	425	1.00	Referent
	1-9 days	107	1.09 (0.77-1.53)	0.63	28	1.25 (0.79-1.96)	0.34
	10-19 days	74	1.13 (0.76-1.66)	0.55	15	0.85 (0.44-1.66)	0.64
	≥20 days	298	1.45 (1.19-1.77)	<0.01	85	1.20 (0.91-1.58)	0.20
Cigarettes Smoked in 30 Days Before OSUT	None	1375	1.00	Referent	425	1.00	Referent
	1-9 cigarettes/day	270	1.29 (1.04-1.59)	0.02	80	1.11 (0.84-1.48)	0.46
	10-19 cigarettes/day	126	1.23 (0.91-1.67)	0.17	31	1.12 (0.71-1.77)	0.61
	≥20 cigarettes/day	66	1.76 (1.23-2.52)	<0.01	17	1.51 (0.88-2.59)	0.13
Used Smokeless Tobacco 30 Days Before OSUT	No	1473	1.00	Referent	536	1.00	Referent
	Yes	365	1.00 (0.82-1.21)	0.96	17	0.80 (0.44-1.45)	0.46
Physical Activity Before OSUT Compared to Peers	Much less active	91	1.52 (1.06-2.17)	0.02	62	1.25 (0.81-1.93)	0.32
	Less active	315	1.53 (1.20-1.96)	<0.01	128	1.14 (0.78-1.67)	0.49
	Average	441	1.08 (0.85-1.38)	0.53	146	0.90 (0.62-1.31)	0.58
	More active	637	0.85 (0.67-1.08)	0.18	161	0.84 (0.57-1.22)	0.35
	Much more active	354	1.00	Referent	56	1.00	Referent
Exercise or Sports Frequency 2 Months Before OSUT	≤1 time/week	281	1.42 (1.12-1.80)	<0.01	90	1.39 (1.02-1.91)	0.04
	2-4 time/week	912	0.97 (0.82-1.15)	0.70	310	1.13 (0.88-1.44)	0.34
	≥5 time/week	707	1.00	Referent	153	1.00	Referent
Time Running/Jogging before OSUT	≤1 month	451	1.38 (1.08-1.68)	<0.01	143	1.66 (1.18-2.32)	<0.01
	2-6 months	949	1.06 (0.87-1.30)	0.09	308	1.63 (1.20-2.21)	<0.01
	≥7 months	437	1.00	Referent	102	1.00	Referent
Time Weight Training before OSUT	≤1 month	660	1.25 (1.04-1.51)	<0.01	274	1.47 (1.08-2.01)	0.02
	2-6 months	537	1.12 (0.91-1.36)	0.11	190	1.33 (0.95-1.86)	0.09
	≥7 months	640	1.00	Referent	88	1.00	Referent
Prior Lower Limb Injury	No	1327	1.00	Referent	406	1.00	Referent
	Yes	510	1.19 (1.01-1.41)	0.04	147	1.08 (0.85-1.35)	0.54
Prior Injury Prevent Activities ≥1 Week	No prior injury	1327	1.00	Referent	406	1.00	Referent
	No	171	1.12 (0.92-1.38)	0.26	45	1.07 (0.72-1.58)	0.38
	Yes	337	1.29 (1.00-1.67)	0.05	102	1.22 (0.79-1.88)	0.75
Totally Recovered from Prior Injury	No prior injury	1325	1.00	Referent	406	1.00	Referent
	No	27	2.07 (1.22-3.52)	<0.01	11	1.50 (0.77-2.92)	0.23
	Yes	481	1.15 (0.97-1.37)	0.11	136	1.05 (0.82-1.33)	0.71
Age at Menarche	No menses yet				4	0.23 (0.03-1.67)	0.15
	6-10 years				48	1.21 (0.85-1.72)	0.29
	11-14 years				437	1.00	Referent
	15-17 years				64	0.78 (0.56-1.10)	0.16
Gone ≥6 Months without Menstrual Cycle in Last Year	No				494	1.00	Referent
	Yes				44	1.60 (1.13-2.26)	<0.01
	No menses yet				4	0.25 (0.04-1.76)	0.16
Taken Birth Control Pills in Last 12 Months	No				299	1.00	Referent
	Yes				249	1.11 (0.90-1.36)	0.33
Ever Pregnant	No				494	1.00	Referent
	Yes				59	1.41 (1.02-1.94)	0.04

Table 4 shows the result of the multivariate Cox regression. Complete data for the multivariate analysis was available on 1831 of the 1838 men (99.6%) and 542 of the 553 women (97.5%). Among the men, independent injury risk factors included older age, having smoked ≥100 cigarettes in the past, a lower physical activity self-rating, and reporting not having recovered from a previous injury. Among the women, independent injury risk factors included older age, having smoked ≥100 cigarettes in the past, a shorter time running/jogging before OSUT, and having gone ≥6 months without a menstrual cycle in the last year.

**Table 4:** Multivariate association between questionnaire variables and injury risk among MP recruits

**Variable**	**Strata**	**N**	**Hazard ratio (95% CI)**	**p-value**
**Men**				
Age	<20.0 years	1080	1.00	Referent
	20.0-24.9 years	623	1.21 (1.02-1.43)	0.03
	25–29.9 years	90	1.17 (0.81-1.67)	0.40
	≥30 years	38	2.29 (1.49-3.53)	<0.01
Smoked ≥100 Cigarettes in Life	No	1271	1.00	Referent
	Yes	560	1.32 (1.12-1.56)	<0.01
Physical Activity Before Basic Training Compared to Peers	Much Less Active	90	1.46 (1.01-2.10)	0.05
	Less Active	313	1.51 (1.18-1.94)	<0.01
	Average	439	1.09 (0.85-1.40)	0.49
	More Active	637	0.84 (0.67-1.07)	0.16
	Much More Active	352	1.00	Referent
Totally Recovered from Prior Injury	No Prior Injury	1324	1.00	Referent
	No	27	1.97 (1.16-3.37)	0.01
	Yes	480	1.18 (0.99-1.41)	0.07
**Women**				
Age	<20.0 years	319	1.00	Referent
	20.0-24.9 years	194	1.25 (1.00-1.55)	0.05
	25–29.9 years	20	1.36 (0.77-2.39)	0.29
	≥30 years	9	2.56 (1.19-5.49)	0.02
Smoked ≥100 Cigarettes in Life	No	396	1.00	Referent
	Yes	146	1.20 (0.95-1.51)	0.10
Time Running/Jogging before Basic Training	≤1 month	142	1.61 (1.14-2.26)	<0.01
	2–6 months	300	1.61 (1.18-2.20)	<0.01
	≤7 months	100	1.00	Referent
Gone ≥6 Months without Menstrual Cycle in Last Year	No	494	1.00	Referent
	Yes	44	1.66 (1.17-2.36)	<0.01
	No Cycles Yet	4	0.27 (0.04-1.92)	0.19

## Discussion

The present investigation was the first to indentify the injury risk and injury risk factors in MP training, and only the second [7] to explore these issues in any type of OSUT training. In past studies of Basic Combat Training, injury incidences have ranged between 14% to 42% for men and 41% to 67% for women [6] while the study of infantry OSUT found that the injury incidence among the men was 46% [7]. This compares with the incidence of 34% and 67% of men and women, respectively, in the present study (CII). Comparisons between injury incidence in this investigation and that of past studies are most appropriately performed using the CII. This index has been used in many recent Basic Combat Training investigations [5,16,17] and is similar to that used in older studies that involved screening of hardcopy medical records [2-4]. The injury incidences in the present investigation are within the ranges of that previously seen for Basic Combat training, albeit on the higher end of the previous studies, but lower than that of infantry OSUT. The early part of MP OSUT was essentially identical to Basic Combat Training and a similar injury rate might be expected in this period. MP training continued for an additional 9 weeks and this additional period increased the time at risk.

In addition to injury incidence, the present investigation also identified a number of factors that put recruits at higher injury risk. Older age was an independent injury risk factor among both men and women and this is in consonance with other studies in Basic Combat Training [3,5,7,18,19] as well as other military and civilian investigations where participants performed similar levels of physical activity [20,21]. The reason for the higher susceptibility to injury in older recruits may have to do with age-related changes in stem cells, declines in fitness, and/or prior injury history. The ability of resident stem cells to initiate and conduct tissue repair declines with age [22] and this could make older individuals more susceptible to overuse-type injuries in which small microtraumas accumulate over time and repair in the older tissue does not keep pace with these repeated microtraumas. It is also possible that repetitive microtraumas, coupled with slower regenerative processes, may also weaken muscle and connective tissues to the point where sudden energy exchanges are more likely to cause acute (traumatic) tissue damage. With regard to fitness, aging results in a loss of muscle mass, muscle strength, muscular endurance, aerobic capacity, and flexibility [23,24]. The loss of aerobic capacity and muscular endurance can begin as early as age 25 [24]. These age-related changes reduce absolute fitness levels and may make injuries more likely since lower fitness has been consistently related to injury [2-4,18,25-31]. With regard to prior injuries, it is possible that older recruits may be more likely to have experienced lower limb injuries in the past that make them more susceptible to injuries in MP training. Prior injuries have been shown to be a risk factor for new injuries in many previous studies [29,32-42]. To examine this hypothesis in the present investigation, self-reported prior injuries were stratified by age. Table 5 shows that in the two younger age groups there was little difference in injury incidence during MP training between those with and without prior lower limb injuries. In the oldest age group, there was a weak trend such that those with prior lower limb injuries had a somewhat higher injury incidence during MP training. Thus, the hypothesis that prior injury may make older recruits more susceptible to injuries in training was only weakly supported here.

**Table 5:** Injuries in MP training stratified by prior lower limb injury and age

**Gender**	**Age**	**Response category**	**N**	**Injured in training (%)**	**Risk ratio (95%CI)**	**p-value**^**a**^
Men	<20.0 Year Olds	No Prior Injury	779	30.2	1.16 (0.96-1.40)	0.13
		Prior Injury	306	35.0		
	20.0-24.9 Year Olds	No Prior Injury	462	36.8	1.02 (0.81-1.29)	0.85
		Prior Injury	162	37.5		
	≥25.0 Year Olds	No Prior Injury	86	39.5	1.26 (0.85-1.89)	0.26
		Prior Injury	42	50.0		
Women	<20.0 Year Olds	No Prior Injury	236	62.7	1.04 (0.87-1.25)	0.68
		Prior Injury	89	65.2		
	20.0-24.9 Year Olds	No Prior Injury	145	69.0	1.02 (0.82-1.25)	0.89
		Prior Injury	50	70.0		
	≥25.0 Year Olds	No Prior Injury	25	64.0	1.37 (0.92-2.03)	0.21
		Prior Injury	8	87.5		

^a^Chi-square statistic.

Beside older age, the present investigation found that recruits who reported cigarette smoking in the past were at higher injury risk. Among the men, those who had started smoking earlier in life or had smoked in the 30 days before Basic Combat Training were also at elevated injury risk and a dose–response was generally evident (i.e., progressively more smoking associated with progressively higher injury risk). Women showed a similar trend with regard to early smoking or recent smoking but the association was weaker than that among the men. Interestingly, 87% of men and 88% of women who reported that they had smoked 100 cigarettes in their lives had also smoked in the 30 days before training. If those who had smoked in the 30 days before training were included in the multivariate analysis in place of those who had smoked 100 cigarettes in their lives, the HRs for the other variables changed little and those smoking in the 30 days before training were still at higher risk (Men: HR (smokers/nonsmokers) = 1.28, 95%CI = 1.07-1.51; women : HR (smokers/nonsmokers) = 1.21, 95%CI = 0.96-1.58).

Cigarette smoking prior to basic training has consistently been associated with increased injury risk in US Army and Air Force basic training [3,7,31,43-45] and in the basic training of other countries [18,46]. Further, smoking was associated with injury in infantry soldiers [47] and in other occupational groups [48-50]. Past basic training studies [3,7,18,31,43,44] have also demonstrated a dose–response.

The association between smoking and injuries has biological plausibility, both from a physiological and psychosocial perspective. There is considerable literature showing that cigarette smoking impairs wound [51-58] and bone [59-63] healing, reduces tissue strength [64-69], and affects immune function. The immune system is important for tissue healing, since macrophages, leukocytes, and lymphocytes regulate various steps in the process and remove or assist in removal of damaged tissue [70-73], such as might be produced by microtrauma. The macrophages of smokers have lower phagocytic activity, lower responsiveness to bacterial challenge, and reduced gene expression of the proinflammatory cytokines, which are important for tissue healing [74-76]. Recruits cease smoking once they enter training but the effects of cigarette smoking on immune function is apparent beyond the length of MP OSUT training [77-83]. Besides physiological mechanisms, psychosocial factors can also be considered. Prior studies show that Air Force recruits[84] and civilians [85-87] who were cigarette smokers had higher scores than nonsmokers on various measures of risk taking. Heavy smoking (≥ 20 cigarettes/day) was much more likely to be associated with multiple risk behaviors [87]. It is possible that the higher risk-taking behavior of smokers manifests itself in the activities of basic training and results in a higher injury rate among smokers.

A lower frequency of recent physical activity (exercise or sports) or a shorter history of running or weight training activity was associated with higher injury risk among both men and women. The present data are in consonance with previous studies of military basic training that found increased risk of injury among those who self-reported less physical activity [2,3,6,7,28,88]. In MP training, recruits perform weight-bearing physical activity primarily in the form of standing in formation, walking, and running. It seems reasonable that a higher frequency of weight-bearing physical training prior to training would result in less susceptibility to injury because of the favorable influences of physical activity on the body. Physical activity of the proper intensity, frequency, and duration can increase aerobic fitness, muscle strength, connective tissue strength, and bone health [89,90]. These and other factors may assist in reducing the susceptibility to injury among recruits who were physically active prior to MP training [91].

Men who reported a prior lower limb injury were at higher injury risk. This relationship appeared to be “graded”, depending on the reported severity of the previous injury. That is, recruits reporting at least a week-long limitation of the previous injury were at higher risk in MP training than those who had previous injury but did not report a week-long limitation; those who reported that they had not totally recovered from the previous injury where at much higher risk than those with a prior injury who had recovered. Among the women, the same trends were present but the associations were much weaker, likely because of the smaller sample size. Other studies of basic training have not demonstrated a consistent relationship between prior injuries and injuries in training [5-7,92], although this relationship has often been demonstrated in athletes [35-38,40-42,93]. Some authors have speculated that contractile or connective scar tissues may alter movement mechanics, or that muscle tissue atrophy induced by some injuries might reduce strength or result in muscle imbalances that could affect injuries [94,95]. Many injuries may be chronic or recurrent, accounting for at least a part of this relationship.

Women who reported missing ≥6 or more menstrual periods in the last year were at higher injury risk and this was an independent injury risk factor in the multivariate Cox regression. In past basic training studies, menstrual irregularities have also been shown to increase injury risk [29,43,96]. Investigations of female athletes have also indicated that those with menstrual irregularities have a higher injury incidence of musculoskeletal injuries [97], especially with bone stress injuries [97-99], and that these athletes take longer to recover from their injuries [100]. It has been hypothesized that amenorrhea results in hormonal changes, especially lower estrogen levels, which leads to a reduction in bone mineral density and increasing likelihood of fracture [97,99-101]. Bennell et al. [102] cautioned that athletes with menstrual disturbances also have other risk factors like greater training loads, lower calcium intake, and differences in soft tissue composition. While this may be the case among athletes, in Basic Combat Training the training load is similar for all recruits and all recruits have access to the same calcium sources in the dining facility. Nonetheless, in a Basic Combat Training study in 1993, calcium intake of recruits was only 73 percent of the military recommended daily allowance [4]. One study found that amenorrheic women had lower bone mineral density even after controlling for calcium intake [99].

In the present investigation, a prior pregnancy was associated with higher injury risk but past studies in basic training have demonstrated mixed results regarding this association [27,43]; no other studies could be found in the civilian literature on the effects of prior pregnancy on injuries in physically active women. The American College of Obstetricians and Gynecologists noted that “many of the physiological and morphological changes of pregnancy persist 4–6 weeks postpartum” and recommended that “prepregnancy exercise routines may be resumed gradually as soon as it is medically safe” [103]. One possible mechanism to explain a possible association between prior pregnancy and injury may be the effects of pregnancy on joint laxity. During pregnancy, relaxin acts in concert with estrogen to increase ligament laxity by reducing the density of collagen fiber bundles [104]. This could increase the likelihood of ligament injury due to excessive joint flexibility [105,106]**.** However, the highest levels of relaxin occur in the first trimester and relaxin levels decline for the rest of pregnancy with no antepartum surge, although it continues to be released by the corpus luteum throughout pregnancy [104]. Joint relaxation in the symphysis pubis increases during pregnancy but returns to baseline 3 to 5 months post delivery [106]. Thus, it seems unlikely that joint laxity accounts for the relationship between prior pregnancy and injury, although some longer-term effects of pregnancy cannot be altogether ruled out [106]. It also seemed possible that prior pregnancy covaried with age, because those who had been pregnant were slightly older than those who had not (ages = 20.1 ± 2.1 versus 23.2 ± 5.2, p < 0.01). However, stratifying pregnancy history by age showed little difference in injury incidence during MP training among the age groups for those who had and had not been previously pregnant.

## Conclusions

The present investigation was the first to identify injury rates and identify specific factors increasing injury risk during MP training. At least one training injury was experienced by 34.2% of the men and 66.7% of the women. Recruits were at higher injury risk if they reported that they were older, had smoked in the past, or had performed less frequent exercise or sports prior to MP training. Men were at higher injury risk if they reported a prior injury and women were at higher risk if they reported missing at least six menstrual cycles in the last year or had previously been pregnant.

## Abbreviations

MP: Military Police; OSUT: One-Station Unit Training; AFHSC: Armed Forces Health Surveillance Center; ICD-9: International Classification of Diseases, Revision 9, Clinical Modification; CII: Comprehensive injury index; BMI: Body mass index; SPSS: Statistical Package for the Social Sciences; 95%CI: 95% Confidence interval; HR: Hazard ratio; AWOL: Absent without leave

## Competing interests

The authors declare that they have no competing interests.

## Authors’ contributions

JJK participated in the design of the study, assisted with data collection, assisted with compiling the data, performed the statistical analysis, interpreted the data, and drafted the manuscript. BG, JC, DT, RS collected and complied data, assisted with the interpretation of the data, and helped draft the manuscript. BHJ participated in the design of the study, assisted in the interpretation of the data, and helped draft the manuscript. All authors read and approved the final manuscript.

## Pre-publication history

The pre-publication history for this paper can be accessed here:

http://www.biomedcentral.com/1471-2474/14/32/prepub
